# Place of death and healthcare utilisation at the end of life among individuals with mental and behavioural disorders as underlying cause of death: population-level multiple-register study

**DOI:** 10.1192/bjo.2024.821

**Published:** 2025-01-10

**Authors:** Maja Magdalena Olsson, Christopher Holmberg, Cecilia Larsdotter, Stina Nyblom, Joakim Öhlén

**Affiliations:** Department of Nursing Science, Sophiahemmet University, Stockholm, Sweden; Institute of Health and Care Sciences, University of Gothenburg, Gothenburg, Sweden; Department of Psychotic Disorders, Sahlgrenska University Hospital, Gothenburg, Sweden; Institute of Medicine, Sahlgrenska Academy, University of Gothenburg, Gothenburg, Sweden; and Palliative Centre, Sahlgrenska University Hospital, Gothenburg, Sweden; Institute of Health and Care Sciences, and Centre for Person-centred Care (GPCC) Sahlgrenska Academy, University of Gothenburg, Gothenburg, Sweden; Palliative Centre, Sahlgrenska University Hospital, Gothenburg, Sweden

**Keywords:** Mental and behavioural diseases, severe mental illness, place of death

## Abstract

**Background:**

Understanding the place of death for individuals with mental and behavioural disorders (MBDs) is essential for identifying disparities in healthcare access and outcomes, as well as addressing broader health inequities within this population.

**Aims:**

To examine the place of death among individuals in Sweden with the underlying cause of death reported as a MBD and compare variations between diagnostic groups, as well as explore associations between place of death and individual, sociodemographic and clinical factors.

**Method:**

This population-level analysis used death certificate data (gender, age, underlying cause of death and place of death) recorded between 2013 and 2019 and other national register data. MBD group differences were compared using chi-square tests (χ^2^) and multinominal logistic regressions explored variations in place of death.

**Results:**

The final sample consisted of 2875 individuals. Our regression model revealed that individuals with MBDs other than intellectual disabilities were less likely to die in hospitals (odds ratio 0.60–0.134 [95% CI = 0.014–0.651]) or care homes (odds ratio 0.11–0.97 [95% CI = 0.003–0.355]) than at home. Substance use disorders were the most common underlying cause of death (61.3%). This group consisted predominantly of men (78.8%, χ^2^, *P* < 0.001), and tended to be younger (χ^2^, *P* < 0.001).

**Conclusions:**

Individuals with intellectual and developmental disabilities are more likely to die in hospitals or care homes than at home. Those with MBDs, particularly substance use disorders, face a high risk of premature death, highlighting gaps in healthcare and palliative care provision for these populations.

Mental and behavioural disorders (MBDs) are characterised by the presence of clinical symptoms that impair individuals’ cognitive, emotional and behavioural functions.^1^ Globally, one in eight people are affected by MBDs, which includes substance use disorders, psychotic disorders, affective disorders and intellectual and developmental disabilities.^1–3^ The clinical presentation of MBDs varies across diagnoses. For example, intellectual and developmental disabilities are denoted by limitations in cognitive functioning and adaptive behaviours.^3^ Other MBDs are characterised by delusions, disorganised thoughts and mood alterations, namely, persistent psychotic disorders (e.g. schizophrenia), bipolar disorder and persistent depressive disorders.^1,2^

## Mortality and healthcare gaps in mental and behavioural disorders

Individuals with MBDs tend to have reduced lifespan, with mortalities as much as 20–25 years earlier than the general population.^4–6^ The risk of premature death can be contributed to both chronic somatic conditions, such as cardiovascular and respiratory diseases,^5,7^ as well as suicide.^7^ There are several reasons why individuals with MBDs have poorer physical health, for example, lifestyle-related and behavioural risk factors (e.g. smoking, obesity), side effects of psychotropic medications and the fact that this group to a lower extent receive adequate healthcare, for example, medical and surgical interventions.^8^ Individuals with various MBDs have different healthcare needs but it has been reported that this group generally have limited access to and receive suboptimal healthcare for their conditions.^1,2,9^

## Palliative care challenges

Although individuals with MBDs often die prematurely and may have pronounced palliative care needs, those needs are rarely identified or assessed, indicating potential inequity at the end of life for this group.^10,11^ Palliative care provision for individuals with MBDs has been described as challenging owing to communication barriers ^11,12^ and lack of resources and training in how to care for patients with complex mental health issues.^11,13^ Thus, it has been argued that the identification of palliative care needs for vulnerable populations is limited, as contemporary palliative care practices are founded in caring for individuals dying of cancer.^14^

A widely recognised indicator reflecting the provision of palliatively oriented care is place of death.^15,16^ For individuals with MBDs, place of death has been reported to be contradictory, with large variability across diagnoses, and studies presenting different places as most common place of death (hospitals, own home, care home and other places).^15,17,18^ Thus, gaining population-based knowledge about place of death for people with MBDs as a prerequisite for appropriate palliative care is important to better understand health inequities, such as healthcare access and outcomes.^19^

This study aims to examine the place of death among individuals in Sweden with the underlying cause of death reported as a MBD and compare variations between diagnostic groups, as well as explore associations between place of death and individual, sociodemographic and clinical factors.

## Method

### Study design

This study was part of a larger population-based register study that explored trends in place of death in the general population in Sweden and across different subgroups (e.g. the general population, cancer).^20^

### Variables

Data was obtained from Statistics Sweden, the National Board of Health and Welfare and the Swedish Register for Palliative Care (SRPC). The primary dependent variables were place of death, with four categories: hospital, own home, care home (e.g. nursing home or care facility for individuals with severe and/or chronic disabilities) and other or unknown places (e.g. public places, roads, workplace). The category, ‘other places or unknown’ was omitted in the logistic regressions analyses, as it contained few observations (i.e. posed a risk of disrupting statistical robustness) and would be difficult to interpret inferentially. The registered place of death was identified by the personal identity number of deceased individuals that in turn was linked to the previously mentioned registers in which this information was obtained.

The primary independent variable was the one reported as the underlying cause of death, classified according to the ICD-10.^21^ As the underlying cause of death refers to the disease, injury or circumstances initiating the chain of events leading to the death, it is reported using a single ICD code. Thus, while there might be other conditions contributing or directly leading to the death, these are not included (e.g. intentional or unintentional self-harm and/or comorbidities). However, the underlying cause of death is important, as effective public health interventions should aim to break the chain leading to death. In the context of public health, understanding the underlying cause of death and properly defining and separating it from the (direct) cause of death is important, as it may assist in identifying the preventable and underlying factors contributing to mortality.^22^ This information can inform public health interventions to target specific links or mechanisms in the chain of events leading to death by, for example, focus on prevention, treatment or harm reduction strategies. Deaths registered as ICD-10 codes F00–F99, which relate to MBDs, were grouped into the following categories: substance use disorders, psychotic disorders, bipolar disorders, depression and depressive disorders and intellectual and developmental disabilities.

Individual factors included gender (male/female), age (years) and born in Sweden (yes/no). Variables reflecting socioeconomic factors known to influence the place of death were marital status (unmarried/married/widowed/divorced), single-person household (yes/no), having children under 18 years (yes/no) and educational attainment as categorised by the Swedish classification system SUN2000 (no formal or elementary education, lower secondary education, upper secondary education and higher education). Additional healthcare variables related to individuals’ healthcare utilisation during the month before death, for example, emergency department visits, hospital transfers, cared for in non-specialised or specialised palliative care services and the ICD-code Z51.5 (encounter for palliative care), were also included.

The degree of urbanisation of the deceased's area of residence was reflected by distinguishing between urban and rural areas. Urban areas were defined as regions with continuous settlements or houses, with a maximum distance of 200 metres between houses and a minimum population of 200 inhabitants. A variable reflecting geographical healthcare regions was also included, divided into six categories.

### Sampling and procedure

All deceased adults (≥18 years old) in Sweden who had age, underlying cause of death and place of death recorded in their death certificates between the years 2013 and 2019 were included. The year 2013 was selected as the starting point, as a previous population study exploring place of death had already covered 2012.^23^ In 2012, Sweden also launched and implemented national clinical practice guidelines for palliative care on a national level. The year 2019 was selected to mitigate any potential confounding issues arising from deaths and the issuance of death certificates during the SARS-CoV-2 pandemic.

**Fig. 1:**
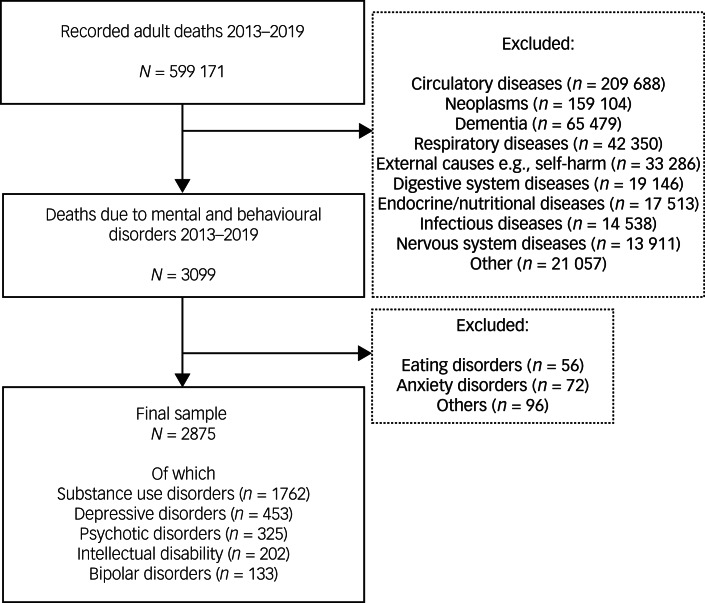
Illustration of sampling procedure, starting with all adults who had an underlying cause of death and place of death recorded in their death certificate between 2013 and 2019.

Between 2013 and 2019, a total of 599 171 death certificates with an underlying cause of death were recorded for adults (≥18 years). Of these, the underlying cause was reported to be a MBD in 3099 people (0.5%). As seen in Supplementary material Table 1 available at https://doi.org/10.1192/bjo.2024.821, no significant annual changes were observed in terms of total deaths, nor in the proportion of deaths attributed to a MBD. After excluding MBD groups that were too rare and thus not suitable for comparative statistical analyses (e.g. eating disorders) the final sample size constituted 2875 people (Fig. 1). Potential changes in deaths and the stability of death certificate issuances in the final sample were evaluated by conducting annual comparisons. No major differences were observed (Supplementary material Table 2).

### Statistical analyses

The included variables contained less than 5% missing data. Thus, statistical analyses were performed on all available data. Descriptive statistics were employed to analyse frequencies and percentages between MBD groups and statistically compare their distributions using chi-square tests (χ^2^). We initiated our analyses by conducting univariable multinominal logistic regressions to explore the disparities in the place of death among people dying from different MBDs (Supplementary Table 3). This allowed us to analyse each variable in isolation, discerning its individual impact on the dependent variables (place of death). Odds ratios were calculated with their 95% confidence intervals. Associations were evaluated using omnibus *F*-tests, based on the difference in −2 log-likelihoods between the final model and a reduced model (generated by omitting one effect from the final model).

Lastly, multivariable logistic regression models were performed. Multicollinearity was assessed by evaluating pairwise correlation coefficients between independent variables, using *r_s_* = >0.70 as a cut-off.^24^ In model 1, individual variables were included (gender, age). In model 2, cause of death was added (different MBD). In model 3, socioeconomic and an urban/rural factor were added (born in Sweden, educational attainment, marital status, persons in household residing in an urban area). In model 4, healthcare-related factors were added (healthcare region, number of emergency department visits and number of hospital transfers, in the month before death). The models’ goodness-of-fit was assessed using the area under the receiver operating characteristic curve (AUC statistics). For models 1–4 see Supplementary Tables 4–7.

## Results

### Sample characteristics

ICD-10 codes F00–F99 were grouped into larger MBD groups to enable meaningful statistical analyses, namely substance use disorders (F10–F19), psychotic disorders (F20–F29), bipolar disorders (F31), depression and depressive disorders (F32–F39) and intellectual and developmental disabilities (F70–F79). Of all the individuals with a MBD recorded as their cause of death in Sweden between 2013 and 2019, most were attributed to substance use disorders (*n* = 1762, 61%). This was also the group with the lowest median age (median 67, interquartile range (IQR) 15), compared with the oldest group, individuals who died from depression (median 89, IQR 10). Sociodemographic characteristics of the sample segregated by cause of MBD (substance use disorders 61.3%, depression and depressive disorders 15.8%, psychotic disorders 11.3%, intellectual and developmental disabilities 7% and bipolar disorders 4.6%) can be seen in Table 1. The results showed variations in death related to gender across diagnoses where men to a higher extent died from substance use disorders (78.8%) and females from psychotic disorders (64.9%), bipolar disorders (70.7%) and depression and depressive disorders (70.9%). Most were born in Sweden (86.2–97%). Individuals who died of intellectual and developmental disabilities had the highest percentage of not having any formal educational attainment (59.8%). Marital status varied across the sample, individuals with intellectual and developmental disabilities being the least prone to ever having been married (97%). Most individuals lived in single-person households (67.9–79.5%) and the majority did not have a child under the age of 18 in the household (97.1–99.5%). Most individuals who died from MBDs resided in urban areas (90.2–97.5%). There was variability in healthcare region belonging at the time of death whereby the percentage of those dying from substance use disorders was lowest in the south-east region (8.5%) and highest in the west region (27.5%). For individuals who died of psychotic disorders, the south-east region had the lowest percentage (12%) and Uppsala-Örebro region the highest (23.1%). Deaths from bipolar disorders were least common in the north and south-east region (10.5%) and highest in the Uppsala-Örebro region (25.6%). The percentage of individuals dying from depression was lowest in the north region (6%) and highest in the west region (33.1%). Finally, deaths caused by intellectual and developmental disabilities were least common in the Stockholm region (7.4%) and highest in the Uppsala-Örebro region (24.3%).

**Table 1:** Sociodemographic characteristics of the sample

	*N*	Substance use disorders	Psychotic disorders	Bipolar disorders	Depression and depressive disorders	Intellectual and developmental disabilities	χ^2^ (*P*)
Age at death, years	2875						1309.183 (<0.001)
18–39, *n* (%)		57 (3.2)	1 (0.3)	0 (0.0)	0 (0.0)	5 (2.5)	
40–59, *n* (%)		409 (23.2)	15 (4.6)	2 (1.5)	2 (0.4)	44 (21.8)	
60–69, *n* (%)		594 (33.7)	50 (15.4)	14 (10.5)	10 (2.2)	61 (30.2)	
70–79, *n* (%)		492 (27.9)	88 (27.1)	40 (30.1)	48 (10.6)	51 (25.2)	
80–89, *n* (%)		185 (10.5)	111 (34.2)	56 (42.1)	190 (41.9)	32 (15.8)	
≥90, *n* (%)		25 (1.4)	60 (18.5)	21 (15.8)	203 (44.8)	9 (4.5)	
Gender	2875						588.045 (<0.001)
Male, *n* (%)		1388 (78.8)	114 (35.1)	39 (29.3)	132 (29.1)	102 (50.5)	
Female, *n* (%)		374 (21.2)	211 (64.9)	94 (70.7)	321 (70.9)	100 (49.5)	
Born in Sweden, *n* (%)	2875	1520 (86.3)	293 (90.2)	128 (96.2)	414 (91.4)	196 (97.0)	35.986 (<0.001)
Educational attainment	2756						388.807 (<0.001)
No formal or elementary education, *n* (%)		352 (20.3)	159 (50.3)	41 (31.1)	230 (51.9)	76 (59.8)	
Lower secondary education, *n* (%)		320 (18.4)	27 (8.5)	21 (15.9)	28 (6.3)	46 (36.2)	
Upper secondary education, *n* (%)		898 (51.7)	105 (33.2)	48 (36.4)	143 (32.3)	5 (3.9)	
Higher education, *n* (%)		168 (9.7)	25 (7.9)	22 (16.7)	42 (9.5)	0 (0.0)	
Marital status	2871						957.235 (<0.001)
Married, *n* (%)		210 (11.9)	15 (4.6)	25 (18.8)	71 (15.7)	1 (0.5)	
Unmarried, *n* (%)		661 (37.6)	161 (49.5)	27 (20.3)	39 (8.6)	196 (97.0)	
Widow, *n* (%)		196 (11.1)	68 (20.9)	46 (34.6)	268 (59.2)	0 (0.0)	
Divorced, *n* (%)		691 (39.3)	81 (24.9)	35 (26.3)	75 (16.6)	5 (2.5)	
Persons in household	2810						13.557 (0.009)
Single-person household, *n* (%)		1214 (70.5)	238 (77.8)	89 (67.9)	326 (72.1)	159 (79.5)	
Multi-person household, *n* (%)		507 (29.5)	68 (22.2)	42 (32.1)	126 (27.9)	41 (20.5)	
Children under 18 in household	2810						10.558 (0.032)
No, *n* (%)		1671 (97.1)	302 (98.7)	129 (98.5)	447 (98.9)	199 (99.5)	
Yes, *n* (%)		50 (2.9)	4 (1.3)	2 (1.5)	5 (1.1)	1 (0.5)	
Residing in urban area	2871						20.133 (<0.001)
Yes, *n* (%)		1585 (90.2)	306 (94.2)	120 (90.2)	425 (93.8)	197 (97.5)	
No, *n* (%)		173 (9.8)	19 (5.8)	13 (9.8)	28 (6.2)	5 (2.5)	
Healthcare region	2871						98.007 (<0.001)
North region, *n* (%)		216 (12.3)	42 (12.9)	14 (10.5)	27 (6.0)	32 (15.8)	
Uppsala-Örebro region		305 (17.3)	75 (23.1)	34 (25.6)	83 (18.3)	49 (24.3)	
Stockholm region		250 (14.2)	70 (21.5)	24 (18.0)	73 (16.1)	15 (7.4)	
West region		484 (27.5)	47 (14.5)	27 (20.3)	150 (33.1)	37 (18.3)	
South-east region		150 (8.5)	39 (12.0)	14 (10.5)	54 (11.9)	26 (12.9)	
South region		353 (20.1)	52 (16.0)	20 (15.0)	66 (14.6)	43 (21.3)	

### MBDs, place of death and healthcare utilisation before time of death

An overview of the differences in place of death and healthcare utilisation before time of death for individuals with MBDs is presented in Table 2. The most common place of death for all MBD groups was nursing homes or care facilities (69.2–78.2%). The only exception was for those dying from substance use disorders, who were more likely to die at home (51.5%). Approximately two in ten individuals who died of MBDs had one or more visits to the emergency department during their last month of life, and for individuals who died from bipolar disorder, this number was nearly three in ten, although the differences were not statistically significant (*P* = 0.102). Few individuals died in specialised palliative care settings (0.3–3.0%), and only a small number had the ICD code Z51.5 (encounter for palliative care) recorded (0.6–3.8%).

**Table 2:** Mental and behavioural disorders, place of death and healthcare utilisation before time of death

	*N*	Substance use disorders	Psychotic disorders	Bipolar disorders	Depression and depressive disorders	Intellectual and developmental disabilities	χ^2^ (*P*)
Place of death	2875						792.311 (<0.001)
Hospital, *n* (%)		344 (19.5)	47 (14.5)	30 (22.6)	51 (11.3)	43 (21.3)	
Nursing or care home, *n* (%)		459 (26.0)	254 (78.2)	92 (69.2)	351 (77.5)	150 (74.3)	
Home, *n* (%)		907 (51.5)	24 (7.4)	11 (8.3)	50 (11.0)	7 (3.5)	
Other places or unknown, *n* (%)		52 (3.0)	0 (0.0)	0 (0.0)	1 (0.2)	2 (3.0)	
Emergency department visits the month before death	2875						13.298 (0.102)
None, *n* (%)		1428 (81.0)	250 (76.9)	97 (72.9)	366 (80.8)	153 (75.7)	
One or more visit, *n* (%)		334 (19.0)	75 (23.1)	36 (27.1)	87 (19.2)	49 (24.3)	
Hospital transfers the month before death	2875						27.769 (<0.001)
None, *n* (%)		1355 (76.9)	233 (71.7)	82 (61.7)	318 (70.2)	141 (69.8)	
One, *n* (%)		265 (15.0)	60 (18.5)	32 (24.1)	96 (21.2)	44 (21.8)	
Two or more, *n* (%)		142 (8.1)	32 (9.8)	19 (14.3)	39 (8.6)	17 (8.4)	
Place of death within specialised palliative care	2875						17.172 (0.002)
No, *n* (%)		1756 (99.7)	324 (99.7)	129 (97.0)	449 (99.1)	201 (99.5)	
Yes, *n* (%)		6 (0.3)	1 (0.3)	4 (3.0)	4 (0.9)	1 (0.5)	
Encounter for palliative care (ICD-10: Z51.5)	2875						17.285 (0.002)
No, *n* (%)		1751 (99.4)	321 (98.8)	128 (96.2)	443 (97.8)	200 (99.0)	
Yes, *n* (%)		11 (0.6)	4 (1.2)	5 (3.8)	10 (2.2)	2 (1.0)	

### Place of death and associations with socioeconomic, regional and healthcare utilisation

The final multivariable multinominal logistic regression model showed that individuals who died from any MBD other than intellectual disabilities were less likely to die in hospitals (odds ratio 0.60–0.134) or nursing or care homes (odds ratio 0.11–0.97) than at home (Table 3). It was also revealed that a lower age (18–69 years) was associated with a lower likelihood of dying in hospital (odds ratio 0.076–0.291) or a nursing or care home (odds ratio 0.013–0.112) than at home. Having lower or higher secondary attainment were associated with higher likelihood of dying in hospital (odds ratio 1.096–1.766) or in a nursing or care home (odds ratio 1.639–1.543) than at home. Individuals who died from MBDs and had lived in a multi-person household were associated with a higher likelihood of dying in hospitals (odds ratio 2.521) and nursing or care homes (odds ratio 1.437) versus home. Residing in a rural area was associated with a lower likelihood of dying in hospital (odds ratio 0.605) or in a nursing or care home (odds ratio 0.462) than dying at home. Moreover, it was shown that individuals with MBDs who were transferred to hospital in the month before death were less likely to die in hospitals (odds ratio 0.011) or nursing or care homes (odds ratio 0.286) than at home, when compared to those who had no transfer.

**Table 3:** Final model (model 4); multivariable multinomial logistic regression analyses of factors associated with the likelihood of dying in *hospital* versus *home* or in *nursing or care home* versus *at home*

	Hospital versus home			Care home versus home			
		95% CI for odds ratio			95% CI for odds ratio		
	Odds ratio	Lower bound	Upper bound	Odds ratio	Lower bound	Upper bound	*F*-test
Gender							
Male	1						0.594
Female	0.967	0.665	1.406	1.117	0.845	1.477	
Age at death							
18–39	0.298	0.076	1.164	0.013	0.002	0.101	
40–59	0.401	0.157	1.027	0.042	0.021	0.084	
60–69	0.687	0.291	1.621	0.195	0.112	0.341	
70–79	1.288	0.567	2.925	0.725	0.428	1.227	
80–89	1.486	0.674	3.274	1.388	0.844	2.283	
≥90	1						<0.001
Mental health and behavioural disorder							
Substance use disorders	0.060	0.015	0.236	0.011	0.003	0.039	
Psychotic disorders	0.134	0.031	0.575	0.097	0.026	0.355	
Bipolar disorders	0.134	0.028	0.651	0.062	0.015	0.252	
Depressive disorders	0.060	0.014	0.260	0.043	0.012	0.159	
Intellectual disability	1						<0.001
Born in Sweden							
Yes	1						0.913
No	1.037	0.623	1.726	0.944	0.658	1.354	
Educational attainment							
No formal or elementary education	0.791	0.459	1.365	1.075	0.718	1.610	
Lower secondary education	1.096	0.607	1.979	1.639	1.069	2.514	
Upper secondary education	1.766	0.936	3.333	1.543	0.953	2.498	
Higher education	1						0.002
Marital status							
Married	1.777	1.024	3.085	0.804	0.521	1.243	
Unmarried	1.553	1.023	2.358	1.136	0.846	1.524	
Widow	1.210	0.712	2.059	0.981	0.682	1.411	
Divorced	1						0.070
Persons in household							
Single-person household	1						<0.001
Multi-person household	2.521	1.709	3.717	1.437	1.065	1.940	
Residing in urban area							
Yes	1						0.002
No	0.605	0.341	1.073	0.462	0.298	0.714	
Healthcare region							
North region	0.947	0.527	1.701	0.808	0.533	1.227	
South region	0.837	0.503	1.391	0.484	0.333	0.703	
Stockholm region	1.036	0.600	1.789	1.065	0.712	1.593	
West region	0.610	0.376	0.990	0.677	0.482	0.951	
South-east region	1.573	0.852	2.905	1.196	0.761	1.882	
Uppsala-Örebro region	1						<0.001
Emergency department visits the month before death							
None	0.881	0.549	1.414	0.939	0.600	1.471	
One or more visits	1						0.865
Hospital transfers the month before death							
None	0.011	0.007	0.019	0.286	0.179	0.457	
One or more	1						<0.001
Goodness of fit							
Area under ROC curve (95% CI)	0.94 (0.93–0.96)			0.90 (0.89–0.91)			

ROC, receiver operating characteristic.

## Discussion

This study explored where people with mental MBDs die and how it varies across diagnostic groups, while also investigating associations with personal and clinical factors. The research findings revealed that across MBDs, the most common place of death was nursing or care homes (69.2–78.2%) with the exception of those dying from substance use disorders, who more frequently died at home (51.5%). Compared to the general population those numbers are quite high, as the place of death for the general population usually is hospitals (39.2%), nursing or care homes (38%) and at home (20.4%) according to a nationwide Swedish study (which used a similar methodology as our study).^20^ Fewer individuals with substance misuse problems tend to reside in nursing or care homes compared to those with more serious mental illnesses (SMIs), such as schizophrenia.^25^ Schizophrenia is characterised by a more severe psychopathological profile and more significant functional impairments, leading many affected individuals to live in nursing and care homes or receive substantial support services.^25^ In contrast, individuals with substance misuse issues more commonly live in independent housing arrangements. Another explanation for the higher proportion of substance use disorder-related deaths occurring at home might be an underreporting of suicides in home settings.^26^ This underreporting may stem from the ambiguity of recording suicide as an underlying cause of death when the intentional nature of the act is uncertain. Even though suicide is not an underlying cause of death but a direct cause of death, this could be linked to the ‘post-event connection’ not being facilitated, meaning whether the person's suicide was because of an underlying cause such as, for example, alcoholism. This aligns with the abovementioned argument that individuals with substance use issues more frequently live in their ‘own’ homes, perhaps alone, and potentially receive fewer support services from home care, home healthcare, etc., compared to individuals with more severe, chronic debilitating mental illnesses such as schizophrenia. This could also result in more significant underreporting of home as the place of death, as they lack regular visits from support personnel. In Sweden, individuals with severe mental illness (e.g. schizophrenia) often receive more structured support from social services and healthcare, which can lead to more accurate documentation of deaths. In contrast, individuals with substance use disorders who prefer to live independently may not receive the same level of monitoring, potentially leading to underreporting of home deaths. This distinction is important for understanding the context of the findings. A high number of deaths occurring at home (in connection with substance misuse) is therefore not a necessarily positive statistic when put into the perspective of the ‘common’ discourse on palliative care and the place of death, as it is challenging to know what a desirable scenario may be in regard to preferred place of death.

Living in their ‘own’ homes increases both the ‘ambiguity’ and the lack of observers to what has happened, as well as the ability to ‘rescue’ the person in question who experiences an acute event (loneliness).

The multivariable multinomial logistic regression model showed that individuals who died of any MBD other than intellectual and developmental disabilities were less likely to die in hospitals and care homes than in their own home. Rather than being because of their receiving palliative care in their home as the preferred place of death, the implications are that this patient group may experience barriers to accessibility and provision of appropriate healthcare, including palliative care.^27–29^ Individuals with intellectual and developmental disabilities also often have significant comorbidities requiring continuous medical care, which may explain why they are more likely to reside in care homes long term and less likely to die in hospitals or need emergency care near the end of life. Concerns regarding barriers to accessing appropriate healthcare, including palliative care, for individuals with MBDs stem from broader challenges in healthcare delivery, such as stigma, lack of specialised services and difficulties in communication and understanding of their unique needs, rather than direct evidence of their preferences or care provision. Further research is needed to explore these issues comprehensively. Another plausible explanation is that individuals with MBDs, for example, substance use disorders, prefer independence and choose not to be in care settings, for example, care homes, as they might be prevented from using substances or receive inadequate dosages of analgesics and sedatives.^27^

The results of the study revealed that in Sweden, the most common underlying cause of death among individuals dying from a MBD between 2013 and 2019 was substance use disorders (61.3%), and this group also presented the lowest median age of when death occurred (median 67). This stands in a 16-year contrast to the median life expectancy in Sweden, which was around 83 years in year 2021.^30^ Substance use disorders often lead to various health complications and risky behaviours that can result in premature death, which may be an explanation for the lower age at death.^31,32^ The fact that substance use disorders are associated with premature deaths more often than other MBDs is important, as it indicates a public health issue related to this group's access to healthcare and perhaps also to their palliative care needs being met, with access to specialised palliative care consultation and expertise. It has been reported that individuals with substance use disorders are not adequately included in palliative care efforts, and their voices are not heard, partly because of a lack of knowledge and attention in the field of addiction and palliative care, and partly because of the fragmented organisation of healthcare, which is unable to address their complex medical and psychosocial needs.^27^

For individuals with MBDs, previous research has shown mixed results for place of death, although this group is more likely to die in care homes.^29^ People with MBDs also tend to be vulnerable, with such individuals rarely thought of in terms of palliative care, mainly because it promotes care for individuals with somatic diseases.^10,27,28,33^ Integration of palliative care within mental health settings or homeless shelters has been reported as difficult. The MBD population often experiences prejudice within the healthcare system, lacks acknowledgement of their social determinants of health and is given limited opportunity for psychiatric intervention, the latter neglecting human suffering as a complex issue.^34^ The International Association for Hospice and Palliative Care emphasises to relieve suffering associated with any illness or injury and to address and make those services available and accessible to especially vulnerable populations, thus making it applicable to individuals with MBDs.^35^ The suggestion to include these aspects highlights that, historically, the concept of palliative care has been restricted to severe physical illnesses, something that may be too narrow and might also explain why research on palliative care interventions and strategies is scarce for individuals with MBDs.^10,36^ From a public health perspective, it would be beneficial if palliative care efforts aim to promote equity and access to care for those who need it. However, as palliative care services are limited, provision models are unable to meet people's potential palliative care needs, and this is especially true of individuals from vulnerable groups with additional needs that may not fit into current palliative care models.^10,37^ Hence, there is a need to focus on how to better integrate a palliative care approach into the mental healthcare provided to those with MBDs, for example, by giving access to specialist palliative consultation teams who collaborate with those having expertise in caring for people with MBDs. Moreover, individuals with MBDs are often rather isolated and, for some, dying at home would imply a lonely death. It would be appropriate to see how palliative care can mitigate this.

In our study, individuals with MBDs who were transferred to hospital in the last month before death were less likely to die in hospitals and care homes than in their own homes, compared to those with no transfer. In a population-based study, Fond et al^38^ found that patients with schizophrenia were more likely to receive palliative care in the last month of life and were less likely to undergo aggressive treatments such as chemotherapy and surgery. Sheridan^10^ emphasises that the increased use of palliative care may result from late cancer diagnoses and underscores that palliative care needs are often overlooked when transitioning from curative care or early on in the management of chronic health conditions, thus neglecting quality-of-life issues.

Our study found that individuals with MBDs living in multi-person households were more likely to die in hospitals or care homes than at home. Those in multi-person households may have social networks that can call for medical assistance or transport them to healthcare. Family members may also feel overwhelmed when caring for someone with MBDs and prefer admission to hospital or placement in a care home. In addition, limitations associated with providing home care may lead families to rely on hospitals for support.^39^ Only a small number of individuals had children under the age of 18 in the household. However, the loss of a parent or custodian is typically a traumatic event and underscores the importance of identifying and addressing the need for palliative care to prepare these children for the loss of a parent or custodian.

Living in a rural area was associated with a lower risk of dying in care homes versus at home. In rural areas there is frequently less access to different care organisations than in urban settings, and any resources in rural areas may be focused on providing care and support for individuals in their own home rather than in institutions like care homes. The number of individuals dying from various MBDs in hospitals and care homes differed across regions. A separate Swedish study has shown that regional differences are common, not only in terms of geography (e.g. urban/rural gradient) but also in how accessibility to healthcare services varies across regions.^40^ The present study found great dissimilarity in the number of deaths across regions in Sweden, revealing that regional mortality could not solely be explained by supply and healthcare utilisation, or by underlying medical need and demand.^40^

### Strengths and limitations

Death certificates have long served as a valuable tool in public health policy and as an established research resource. One of the significant advantages of death certificates lies in their comprehensive nature, enabling the examination of patterns across an entire population rather than solely relying on sample data. The coverage rate is also high, and the proportion of missing certificates is only around 1%. Consequently, the study of place of death offers insights that extend beyond specific patient populations and encompasses diverse healthcare settings. We can therefore gain a broader understanding of the distribution and dynamics surrounding end-of-life care and location of death across various contexts.

The use of death certificates also comes with limitations, for example, uncertainty regarding the reliability of the codes for the underlying cause of death. As these codes can be based on different investigation methods it is considered that the more detailed the investigation, the more certain the stated cause of death. The information about cause of death is generally assumed to be more reliable for younger people than for the elderly, and more trustworthy for violent deaths and illnesses with a dramatic course than for chronic conditions. Still, information from death certificates is carefully assessed for accuracy before being recorded in the Swedish Cause of Death Register.^41^ The widespread use of this registry in research studies further attests to its reliability. Another noteworthy limitation of the study is that we did not have any data on visits to primary or out-patient care, or on how much social support individuals received (except for those who lived in care homes), or other social care services and informal care.

Another limitation of the study is the absence of data on comorbidity, or individuals with multiple diagnoses. This is important because the group with MBDs at the end of life could be quite diverse. However, these individuals are often overlooked in discussions regarding the improvement of palliative care. Consequently, there is a risk of marginalisation for this group in various aspects. The authors suggest that future studies undertake retrospective medical record reviews or use prospective study designs to more deeply explore what individuals with MBDs commonly seek treatment for within the healthcare system, what kind of medical or psychiatric issues are identified and what subsequent care is planned for them. Individuals with intellectual and developmental disabilities tend to die in hospitals or care homes rather than at home, even when considering sociodemographic and healthcare utilisation factors. Palliative care needs may not be adequately identified for those with MBDs, especially for individuals with substance use disorders and intellectual and developmental disabilities, which highlights a potential public health concern related to equity in health and healthcare service provision for vulnerable populations. Therefore, increased efforts are needed to recognise and address potential palliative care needs for individuals with MBDs, with an emphasis on those with intellectual and developmental disabilities and substance use disorders, as well as for those with under-age children. Policy-level recognition of the neglect of individuals with MBDs and the need for tailored and additional palliative care support is crucial, requiring further research to better understand their palliative care needs. Overall, there is a general lack of understanding about how to implement palliative care for individuals with psychiatric conditions. This highlights the necessity of adapting palliative care knowledge to make it relevant for those with psychiatric conditions, rather than applying it indiscriminately.

## Supporting information

Olsson et al. supplementary material Olsson et al. supplementary material

## Data Availability

This study used de-identified individual-level data from Swedish healthcare registers that are not publicly available, in accordance with Swedish legislation. The data can be obtained from the respective Swedish data holders on the basis of ethics approval for the research in question, subject to relevant legislation, processes and data protection.
