# Supplementary material for: Place of death and healthcare utilisation at the end of life among individuals with mental and behavioural disorders as underlying cause of death: population-level multiple-register study
Source: BJPsych Open. 2025 Jan 10;11(1):e14. doi: 10.1192/bjo.2024.821 (PMC11733492; doi:10.1192/bjo.2024.821)
Supplement: Olsson et al. supplementary material [file S2056472424008214sup001.docx]

**Supplementary materials**

**Supplementary Table 1.** Number of recorded deaths among adults (≥18 years old) in Sweden (N= 599,171) between 2013-2019, segregated annually, with the proportion of deaths attributed to MBD (N=3,099).

| **Year recorded** | **Total number of recorded deaths** | **Proportion of deaths attributed to MBD (ICD-10 codes: F00-F99).**  **n (% of total number of deaths recorded)** |
| --- | --- | --- |
| 2013 | 83,757 | 382 (0.5) |
| 2014 | 83,063 | 433 (0.5) |
| 2015 | 85,442 | 457 (0.5) |
| 2016 | 86,163 | 439 (0.5) |
| 2017 | 87,804 | 447 (0.5) |
| 2018 | 88,036 | 466 (0.5) |
| 2019 | 84,906 | 475 (0.5) |

| **Supplementary Table 2.** Number of recorded deaths among adults (≥18 years old) in Sweden due to MBD (n= 2,875) between 2013-2019. Data is segregated annually, with proportion of deaths attributed to substance use disorders (n=1,762), psychotic disorders (n=325), bipolar disorders (n=133), depression and depressive disorders (n=453), and intellectual and developmental disabilities (n=202). | | | | | | |
| --- | --- | --- | --- | --- | --- | --- |
| **Year recorded** | **Total number of recorded deaths due to MBD** | **Substance use disorders (%)** | **Psychotic disorders, n (%)** | **Bipolar disorders, n (%)** | **Depressive disorders,**  **n (%)** | **IDD (%)** |
| 2013 | 352 | 198 (56.3) | 41 (11.6) | 18 (5.1) | 68 (19.3) | 27 (7.7) |
| 2014 | 397 | 243 (61.2) | 36 (9.1) | 17 (4.3) | 71 (17.9) | 30 (7.6) |
| 2015 | 411 | 225 (54.7) | 48 (11.7) | 24 (5.8) | 81 (19.7) | 33 (8.0) |
| 2016 | 409 | 241 (58.9) | 43 (10.5) | 19 (4.6) | 71 (17.4) | 35 (8.6) |
| 2017 | 417 | 239 (57.3) | 57 (13.7) | 28 (6.7) | 65 (15.6) | 28 (6.7) |
| 2018 | 444 | 293 (66.0) | 63 (14.2) | 12 (2.7) | 48 (10.8) | 28 (6.3) |
| 2019 | 445 | 323 (72.6) | 37 (8.3) | 15 (3.4) | 49 (11.0) | 21 (4.7) |

**Supplementary Table 3**. Univariable multinomial logistic regression analyses of factors associated with the likelihood of dying in hospital (n=515) versus home (n=999) and dying in a care home (n=1,306) versus at home among adults who had an MBD stated as the underlying cause for their death (N=2,820)

|  |  |  |  |  |  |  |  |  |
| --- | --- | --- | --- | --- | --- | --- | --- | --- |
|  |  | **Hospital vs Home** | | | **Nursing home vs Home** | | | **F-test** |
|  |  |  | 95% CI for OR | |  | 95% CI for OR | |  |
|  |  | OR | Lower bound | Upper bound | OR | Lower bound | Upper bound |  |
| **MBD** | | | | | | | |  |
| Substance use disorders |  | .062 | .028 | .139 | .024 | .011 | .051 |  |
| Psychotic disorders |  | .319 | .125 | .815 | .494 | .208 | 1.174 |  |
| Bipolar disorders |  | .444 | .154 | 1.277 | .390 | .146 | 1.043 |  |
| Depressive disorders |  | .166 | .068 | .404 | .328 | .145 | .739 |  |
| Intellectual and developmental disabilities |  | 1 | | | | | | <.001 |
| **Year of death** | | | | | | | |  |
| 2013 |  | 1.481 | .980 | 2.237 | 1.296 | .949 | 1.770 |  |
| 2014 |  | 1.723 | 1.162 | 2.554 | 1.314 | .970 | 1.780 |  |
| 2015 |  | 1.467 | .979 | 2.199 | 1.520 | 1.127 | 2.049 |  |
| 2016 |  | 1.928 | 1.299 | 2.861 | 1.616 | 1.193 | 2.187 |  |
| 2017 |  | 1.563 | 1.037 | 2.354 | 1.809 | 1.339 | 2.444 |  |
| 2018 |  | 1.499 | 1.020 | 2.203 | 1.178 | .879 | 1.579 | .004 |
| 2019 |  | 1 | | | | | |  |
| **Age at death** | | | | | | | |  |
| 18-39 |  | .873 | .396 | 1.922 | .008 | .002 | .034 |  |
| 40-59, |  | .642 | .357 | 1.154 | .023 | .014 | .036 |  |
| 60-69 |  | .768 | .433 | 1.363 | .077 | .052 | .112 |  |
| 70-79 |  | 1.556 | .870 | 2.785 | .290 | .198 | .426 |  |
| 80-89 |  | 2.102 | 1.113 | 3.968 | .963 | .628 | 1.476 | <.001 |
| ≥90 |  | 1 | | | | | |  |
| **Sex** | | | | | | | |  |
| Male |  | 1 | | | | | |  |
| Female |  | 1.348 | 1.066 | 1.705 | 3.210 | 2.683 | 3.841 | <.001 |
| Born in Sweden |  |  |  |  |  |  |  |  |
| Yes |  | 1 | | | | | |  |
| No |  | .683 | .488 | .958 | .616 | .475 | .798 | <.001 |
| **Educational attainment** | | | | | | | |  |
| No formal education |  | 1.285 | .853 | 1.936 | 3.443 | 2.481 | 4.778 |  |
| Lower secondary education |  | .978 | .647 | 1.479 | .790 | .555 | 1.124 |  |
| Higher secondary education |  | .723 | .501 | 1.043 | .704 | .519 | .956 |  |
| Higher education |  | 1 | | | | | | <.001 |
| **Marital status** | | | | | | | |  |
| Married |  | 3.088 | 2.204 | 4.325 | 1.289 | .949 | 1.750 |  |
| Unmarried |  | 1.526 | 1.179 | 1.974 | 1.275 | 1.046 | 1.554 |  |
| Widow |  | 1.800 | 1.255 | 2.580 | 4.086 | 3.164 | 5.277 |  |
| Divorced |  | 1 | | | | | | <.001 |
| **Persons in household** | | | | | | | |  |
| Single-person household |  | 1 | | | | | |  |
| Multi-person household |  | 2.195 | 1.747 | 2.756 | .879 | .724 | 1.066 | <.001 |
| **Residing in urban area** | | | | | | | |  |
| Yes |  | 1 | | | | | |  |
| No |  | .755 | .529 | 1.079 | .413 | .303 | .564 | <.001 |
| **Health care region** | | | | | | | |  |
| North region, n (%) |  | .938 | .634 | 1.387 | 1.329 | .978 | 1.805 |  |
| Uppsala-Örebro region |  | 1.452 | 1.030 | 2.046 | 2.367 | 1.798 | 3.116 |  |
| Stockholm region |  | 1.956 | 1.366 | 2.800 | 2.522 | 1.869 | 3.401 |  |
| West region |  | .693 | .502 | .958 | 1.208 | .942 | 1.548 |  |
| Southeast region |  | 2.006 | 1.325 | 3.036 | 2.664 | 1.886 | 3.762 |  |
| South region |  | 1 | | | | | | <.001 |
| **ED visits the month before death** | | | | | | | |  |
| None |  | .050 | .037 | .068 | .351 | .262 | .471 |  |
| One or more visits |  | 1 | | | | | | <.001 |
| **Hospital transfers the month before death** | | | | | | | |  |
| None |  | .010 | .007 | .014 | .237 | .174 | .322 |  |
| One or more |  | 1 | | | | | | <.001 |
| CI=Confidence intervals, OR=Odds ratio | | | | | | | | |

| **Supplementary Table 4.** Model 1 (sex, age). Multivariable multinomial logistic regression analyses of factors associated with the likelihood of dying in hospital (n=515) versus home (n=999) and dying in care home (n=1,306) versus at home among adults who had an MBD stated as the underlying cause for their death (N=2,820) | | | | | | | | |
| --- | --- | --- | --- | --- | --- | --- | --- | --- |
|  |  |  |  |  |  |  |  |  |
|  |  | Hospital vs Home | | | Care home vs Home | | | F-test |
|  |  |  | 95% CI for OR | |  | 95% CI for OR | |  |
|  |  | OR | Lower bound | Upper bound | OR | Lower bound | Upper bound |  |
| Sex | | | | | | | |  |
| Male |  | 1 | | | | | |  |
| Female |  | 1.283 | 1.006 | 1.637 | 2.059 | 1.667 | 2.543 | <.001 |
| Age at death | | | | | | | |  |
| 18-39 |  | .973 | .438 | 2.158 | .011 | .003 | .047 |  |
| 40-59 |  | .711 | .392 | 1.289 | .030 | .019 | .048 |  |
| 60-69 |  | .854 | .477 | 1.530 | .104 | .070 | .155 |  |
| 70-79 |  | 1.733 | .959 | 3.131 | .397 | .267 | .589 |  |
| 80-89 |  | 2.231 | 1.178 | 4.225 | 1.136 | .737 | 1.753 |  |
| ≥90 |  | 1 | | | | | | <.001 |
| Goodness-of-fit | | | | | | | |  |
| Area under ROC-curve (95%CI) |  | .618 (.588-.649) | | | 0.83 (0.81–0.84) | | |  |

| **Supplementary Table 5**. Model 2. (+ MBD). Multivariable multinomial logistic regression analyses of factors associated with the likelihood of dying in hospital (n=515) versus home (n=999) and dying in care home (n=1,306) versus at home among adults who had an MBD stated as the underlying cause for their death (N=2,820) | | | | | | | | |
| --- | --- | --- | --- | --- | --- | --- | --- | --- |
|  |  |  |  |  |  |  |  |  |
|  |  | Hospital vs Home | | | Nursing home vs Home | | | F-test |
|  |  |  | 95% CI for OR | |  | 95% CI for OR | |  |
|  |  | OR | Lower bound | Upper bound | OR | Lower bound | Upper bound |  |
| Sex | | | | | | | |  |
| Male |  | 1 | | | | | |  |
| Female |  | .890 | .682 | 1.160 | 1.174 | .921 | 1.498 | .090 |
| Age at death | | | | | | | |  |
| 18-39 |  | 1.703 | .695 | 4.172 | .016 | .003 | .078 |  |
| 40-59 |  | 1.227 | .613 | 2.456 | .044 | .025 | .080 |  |
| 60-69 |  | 1.563 | .795 | 3.073 | .195 | .119 | .318 |  |
| 70-79 |  | 3.035 | 1.550 | 5.946 | .754 | .466 | 1.220 |  |
| 80-89 |  | 2.967 | 1.512 | 5.824 | 1.557 | .965 | 2.514 |  |
| ≥90 |  | 1 | | | | | | <.001 |
| MBD |  | | | | | | | |
| Substance use disorders |  | .050 | .022 | .114 | .013 | .006 | .030 |  |
| Psychotic disorders |  | .254 | .098 | .656 | .152 | .061 | .377 |  |
| Bipolar disorders |  | .309 | .105 | .906 | .082 | .029 | .231 |  |
| Depressive disorders |  | .136 | .053 | .352 | .052 | .021 | .127 |  |
| Intellectual and developmental disabilities |  | 1 | | | | | | <.001 |
| Goodness-of-fit | | | | | | | |  |
| Area under ROC-curve (95%CI) |  | .684 (.654-.713) | | | .882 (.868–.896) | | |  |

| **Supplementary Table 6.** Model 3. (+ born in Sweden, educational attainment, marital status, persons in household, residing in urban area). Multivariable multinomial logistic regression analyses of factors associated with the likelihood of dying in hospital (n=515) versus home (n=999) and dying in care home (n=1306) versus at home among adults who had an MBD stated as the underlying cause for their death (N=2820) | | | | | | | | |
| --- | --- | --- | --- | --- | --- | --- | --- | --- |
|  |  |  |  |  |  |  |  |  |
|  |  | Hospital vs Home | | | Care home vs Home | | | F-test |
|  |  |  | 95% CI for OR | |  | 95% CI for OR | |  |
|  |  | OR | Lower bound | Upper bound | OR | Lower bound | Upper bound |  |
| Sex | | | | | | | |  |
| Male |  | 1 | | | | | | .118 |
| Female |  | .879 | .662 | 1.167 | 1.173 | .902 | 1.526 |  |
| Age at death | | | | | | | |  |
| 18-39 |  | .901 | .328 | 2.473 | .015 | .002 | .117 |  |
| 40-59 |  | .944 | .434 | 2.056 | .044 | .023 | .087 |  |
| 60-69 |  | 1.444 | .691 | 3.018 | .210 | .122 | .361 |  |
| 70-79 |  | 2.993 | 1.464 | 6.121 | .834 | .500 | 1.392 |  |
| 80-89 |  | 3.099 | 1.536 | 6.256 | 1.655 | 1.010 | 2.713 |  |
| ≥90 |  | 1 | | | | | | <.001 |
| MBD |  |  |  |  |  |  |  |  |
| Substance use disorders |  | .047 | .014 | .164 | .012 | .004 | .040 |  |
| Psychotic disorders |  | .218 | .057 | .831 | .123 | .034 | .441 |  |
| Bipolar disorders |  | .244 | .058 | 1.030 | .079 | .020 | .314 |  |
| Depressive disorders |  | .106 | .028 | .407 | .049 | .014 | .174 |  |
| Intellectual and developmental disabilities |  | 1 | | | | | | <.001 |
| Born in Sweden | | | | | | | |  |
| Yes |  | 1 | | | | | |  |
| No |  | .809 | .558 | 1.173 | .908 | .642 | 1.285 | .526 |
| Educational attainment | | | | | | | |  |
| No formal education |  | .854 | .572 | 1.275 | 1.117 | .762 | 1.638 |  |
| Lower secondary education |  | .941 | .599 | 1.478 | 1.617 | 1.078 | 2.425 |  |
| Higher secondary education |  | 1.348 | .849 | 2.138 | 1.503 | .949 | 2.381 |  |
| Higher education |  | 1 | | | | | | .004 |
| Marital status | | | | | | | |  |
| Married |  | 1.989 | 1.337 | 2.957 | .816 | .536 | 1.241 |  |
| Unmarried |  | 1.311 | .970 | 1.772 | 1.139 | .858 | 1.512 |  |
| Widow |  | 1.290 | .847 | 1.965 | .995 | .703 | 1.409 |  |
| Divorced |  | 1 | | | | | | .001 |
| Persons in household |  | | | | | | |  |
| Single-person household |  | 1 | | | | | | <.001 |
| Multi-person household |  | 2.290 | 1.730 | 3.031 | 1.439 | 1.081 | 1.916 |  |
| Residing in urban area | | | | | | | |  |
| Yes |  | 1 | | | | | | .001 |
| No |  | .615 | .408 | .925 | .477 | .315 | .723 |  |
| Goodness-of-fit | | | | | | | |  |
| Area under ROC-curve (95%CI) |  | .73 (.70–.76) | | | .89 (.88–.90) | | |  |

| **Supplementary Table 7.** Model 4 (+Number of ED visits and hospital transfers the month before death). Multivariable multinomial logistic regression analyses of factors associated with the likelihood of dying in hospital (n=515) versus home (n=999) and dying in care home (n=1,306) versus at home among adults who had an MBD stated as the underlying cause for their death (N=2,820) | | | | | | | | |
| --- | --- | --- | --- | --- | --- | --- | --- | --- |
|  |  |  |  |  |  |  |  |  |
|  |  | **Hospital vs Home** | | | **Nursing home vs Home** | | | **F-test** |
|  |  |  | 95% CI for OR | |  | 95% CI for OR | |  |
|  |  | OR | Lower bound | Upper bound | OR | Lower bound | Upper bound |  |
| **Sex** | | | | | | | |  |
| Male |  | 1 | | | | | | .594 |
| Female |  | .967 | .665 | 1.406 | 1.117 | .845 | 1.477 |  |
| **Age at death** | | | | | | | |  |
| 18-39 |  | .298 | .076 | 1.164 | .013 | .002 | .101 |  |
| 40-59 |  | .401 | .157 | 1.027 | .042 | .021 | .084 |  |
| 60-69 |  | .687 | .291 | 1.621 | .195 | .112 | .341 |  |
| 70-79 |  | 1.288 | .567 | 2.925 | .725 | .428 | 1.227 |  |
| 80-89 |  | 1.486 | .674 | 3.274 | 1.388 | .844 | 2.283 |  |
| ≥90 |  | 1 | | | | | | <.001 |
| **MBD** | | | | | | | |  |
| Substance use disorders |  | .060 | .015 | .236 | .011 | .003 | .039 |  |
| Psychotic disorders |  | .134 | .031 | .575 | .097 | .026 | .355 |  |
| Bipolar disorders |  | .134 | .028 | .651 | .062 | .015 | .252 |  |
| Depressive disorders |  | .060 | .014 | .260 | .043 | .012 | .159 |  |
| Intellectual and developmental disabilities |  | 1 | | | | | | <.001 |
| **Born in Sweden** | | | | | | | |  |
| Yes |  | 1 | | | | | | .913 |
| No |  | 1.037 | .623 | 1.726 | .944 | .658 | 1.354 |  |
| **Educational attainment** | | | | | | | |  |
| No formal education |  | .791 | .459 | 1.365 | 1.075 | .718 | 1.610 |  |
| Lower secondary education |  | 1.096 | .607 | 1.979 | 1.639 | 1.069 | 2.514 |  |
| Higher secondary education |  | 1.766 | .936 | 3.333 | 1.543 | .953 | 2.498 |  |
| Higher education |  | 1 | | | | | | .002 |
| **Marital status** |  | | | | | | |  |
| Married |  | 1.777 | 1.024 | 3.085 | .804 | .521 | 1.243 |  |
| Unmarried |  | 1.553 | 1.023 | 2.358 | 1.136 | .846 | 1.524 |  |
| Widow |  | 1.210 | .712 | 2.059 | .981 | .682 | 1.411 |  |
| Divorced |  | 1 | | | | | | .070 |
| **Persons in household** | | | | | | | |  |
| Single-person household |  | 1 | | | | | | <.001 |
| Multi-person household |  | 2.521 | 1.709 | 3.717 | 1.437 | 1.065 | 1.940 |  |
| **Residing in urban area** | | | | | | | |  |
| Yes |  | 1 | | | | | | .002 |
| No |  | .605 | .341 | 1.073 | .462 | .298 | .714 |  |
| **Health care region** | | | | | | | |  |
| North region |  | 1.132 | .631 | 2.030 | 1.672 | 1.090 | 2.565 |  |
| Uppsala-Örebro region |  | 1.195 | .719 | 1.987 | 2.068 | 1.423 | 3.004 |  |
| Stockholm region |  | 1.238 | .720 | 2.130 | 2.202 | 1.460 | 3.320 |  |
| West region |  | .729 | .451 | 1.179 | 1.401 | .987 | 1.988 |  |
| Southeast region |  | 1.880 | 1.021 | 3.461 | 2.474 | 1.558 | 3.928 |  |
| South region |  | 1 | | | | | | <.001 |
| **ED visits the month before death** | | | | | | | |  |
| None |  | .881 | .549 | 1.414 | .939 | .600 | 1.471 |  |
| One or more visits |  | 1 | | | | | | .865 |
| **Hospital transfers the month before death** | | | | | | | |  |
| None |  | .011 | .007 | .019 | .286 | .179 | .457 |  |
| One or more |  | 1 | | | | | | <.001 |
| **Goodness-of-fit** | | | | | | | |  |
| Area under ROC-curve (95%CI) |  | .94 (.93–.96) | | | .90 (.89–.91) | | |  |

¨
